# Supplementary material for: The relation between circulating levels of vitamin D and parathyroid hormone in children and adolescents with overweight or obesity: Quest for a threshold
Source: PLoS One. 2019 Nov 26;14(11):e0225717. doi: 10.1371/journal.pone.0225717 (PMC6879169; doi:10.1371/journal.pone.0225717)
Supplement: Attachment — Submitted filename: Response to Reviewer.docx [file pone.0225717.s002.docx]

**Editor-In-Chief, PLOS ONE**

**Greetings,**

Thank you very much for your email dated October 29^th^, 2019, regarding the secondary evaluation of our manuscript entitled **“The relation between Circulating Levels of Vitamin D and Parathyroid Hormone in children and adolescent with overweight or obesity: Quest for a Threshold (PONE-D-19-15568R1)”** and the opportunity to revise and resubmit the manuscript. We have complied with all reviewer comments, carefully considering each comment, and hope that the revised manuscript is now suitable for publication in *Pols One*. We submit the revised version, with a point by point response (R) to each reviewer comment (C) with changes highlighted.

In the next page, we have addressed the data availability statement and financial disclosure.

Looking forward to hearing from you at your earliest convenience.

Sincerely yours,

**Correspondence to:**

Parvin Mirmiran

Nutrition and Endocrine Research Center, Research Institute for Endocrine Science, Shahid Beheshti University of Medical Sciences, Tehran, Iran

P.O. Box: 19395-4763

Phone: +98 (21) 22432503

Fax: +98 (21) 22402463

E-mail: [mirmiran@endocrine.ac.ir](mailto:mirmiran@endocrine.ac.ir)

Alternative E-mail: [parvin.mirmiran@gmail.com](mailto:parvin.mirmiran@gmail.com)

**Co-correspondence to:**

Farhad Hosseinpanah

Obesity Research Center, Research Institute for Endocrine Sciences, Shahid Beheshti University of Medical Science, Tehran, Iran

P.O. Box: 19395-476

Phone: +98-21-22432500

Fax: +98-21-22416264

E-mail address: [fhospanah@endocrine.ac.ir](mailto:fhospanah@endocrine.ac.ir)

1) **“Availability of data” has been revised as the following:**

The data set is the property of Research Institute for Endocrine Sciences (RIES) and is made available upon approval of the research proposal by the research council and the ethics committee. The RIES ethics committee must issue an approval in case of a request for access to the de-identified dataset. Data request may be sent to the head of the RIES Ethics Committee Dr. Azita Zadeh-Vakili at email: [azitavakili@endocrine.ac.ir](mailto:azitavakili@endocrine.ac.ir).

2) **In the manuscript, we revised the “Funding sources” section as the following:**

*This study was supported by the Shahid Beheshti University of Medical Sciences, Tehran, Iran (Grant no. 10429-4).The funders had no role in study design, data collection and analysis, decision to publish, or preparation of the manuscript. There was no additional external funding received for this study.*

**Response (R) to reviewer comments (C):**

**Reviewer # 1**

1) **I appreciate the revisions to figure 3 to include reference numbers and sample sizes, etc. of each of the studies. There is a typographical error of "Hill MK" instead of "Hill KM".**
2) **Consider use of "sex" or "sexes" rather than "gender" or "genders" as "sex" is biological and "gender" is a social construct.**
3) **line 73 of new text: consider adding "commonly" ("is commonly considered to define")**
4) **line 91 of new text: suggest replacing "impressed" with "affected"**

**R:** Thanks for your profound attention and comment on our manuscript. All above-mentioned concerns have been corrected through the text.
